# Association between the accumulation of elevated serum γ-glutamyltransferase level and risk of atrial fibrillation: a nationwide cohort study

**DOI:** 10.1038/s41598-023-40689-2

**Published:** 2023-08-23

**Authors:** Won Kyeong Jeon, So-Ryoung Lee, Kyung-Do Han, Eue-Keun Choi, Seil Oh, Gregory Y. H. Lip

**Affiliations:** 1 Department of Internal Medicine, Seoul National University Hospital, 101 Daehak-ro, Jongno-gu, Seoul, 03080 Republic of Korea; 2 Department of Internal Medicine, Seoul National University College of Medicine, Seoul, Republic of Korea; 3 Department of Statistics and Actuarial Science, Soongsil University, Seoul, Republic of Korea; 4 Liverpool Centre for Cardiovascular Science, University of Liverpool and Liverpool Chest and Heart Hospital, Liverpool, UK; 5 Department of Clinical Medicine, Aalborg University, Aalborg, Denmark

**Keywords:** Cardiology, Risk factors

## Abstract

Atrial fibrillation (AF) is the most common cardiac arrhythmia. The association between AF and γ-Glutamyltransferase (GGT) was not fully established. This study demonstrated the independent association of cumulative GGT score and AF incidence with the dose-response relationship. Using the Korean National Health Insurance Corporation database, adult subjects who had 4 consecutive annual health examinations from 2009 to 2012 were enrolled. A cumulative GGT score was calculated as the cumulative number of the highest GGT quartile amongst four examinations (0–4 times). A multivariable Cox proportional hazards regression analysis was performed. Among a total of 3,500,847 people included, AF was developed in 27,752 people (0.793%) during a median of 8.0 years of follow up. The incidence rate of AF and adjusted hazard ratio were increased by a stepwise manner in the higher quartile group and cumulative GGT score group. In subgroup analysis, this trend was more prominent in the elderly, people without hypertension, non-obese people, and people without any four comorbidities (diabetes mellitus, hypertension, dyslipidemia, and obesity). Our results suggest multiple accumulation of elevated GGT levels in health examination might be a useful marker for risk stratification of AF development, especially in the elderly and healthy population.

## Introduction

Atrial fibrillation (AF) is the most common cardiac arrhythmia in clinical practice and causes critical complications such as a stroke. The incidence and prevalence of AF are increasing over years in Korea^1^. Many treatment strategies for AF were established, but do not target the underlying causes of AF. The underlying pathogenesis of AF was not fully established, but the evidence regarding AF and oxidative stress was accumulated^2^.

Systemic oxidative stress measured by the redox potentials of glutathione was associated with AF^3^. Antioxidant levels had a negative linear association with serum γ-Glutamyltransferase (GGT) and reverse U-shape association with serum alanine aminotransferase (ALT)^4^. These results suggest that serum GGT may be an early marker of oxidative stress. The ARIC cohort reported that aspartate aminotransferase (AST) and ALT showed a U-shaped association with AF risk^5^. On the contrary, GGT showed linear association, which infer systemic process such as oxidative stress, rather than the hepatic process. Previous studies reported that the GGT level is associated with cardiac arrhythmia, including AF^6–8^. However, these results did not reflect the burden of cumulative oxidative stress during the follow-up period because previous studies were evaluated at only baseline GGT levels.

In this study, we evaluated the relationship between AF and GGT, especially the cumulative burden, in a large population-based cohort.

## Methods

### Data source and study population

The Korean National Health Insurance Service (NHIS) and its database were described severally in former studies^9^. The NHIS covers the entire Korean population, and its claims database includes socio-demographic information, disease diagnosis, medical institute, drugs, and standardized annual medical checkup data. The health checkup data had height, weight, blood pressure, laboratory tests including fasting glucose, cholesterol, AST, ALT, GGT levels, and standardized self-reporting questionnaires about the smoking status and alcohol consumption.

**Figure 1:**
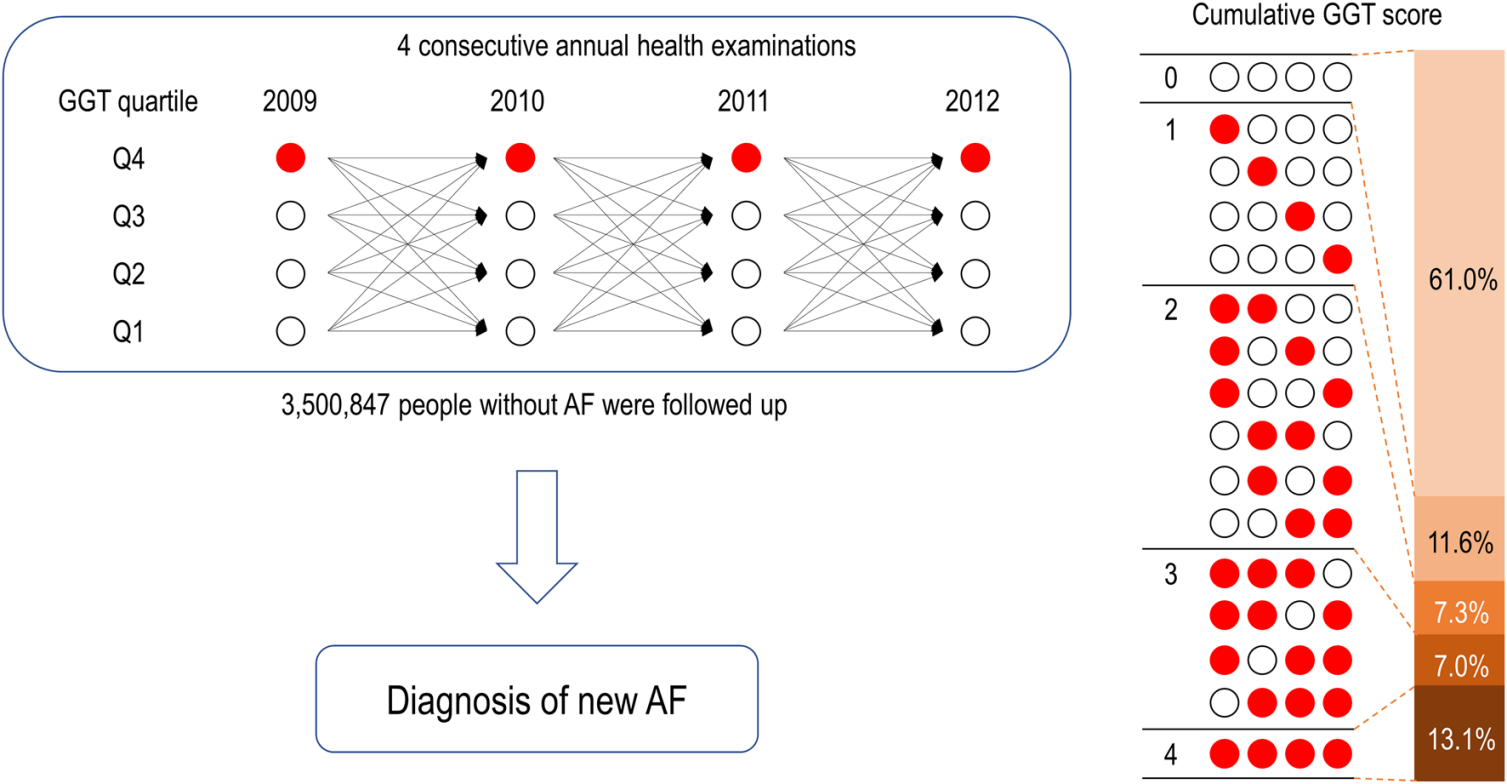
Overall scheme of the study design.

We designed our study to include patients who had their index examination between 2009 and 2012 and had 3 more consecutive examinations before that, so we had 4 consecutive examinations. Of the 23,452,862 people aged over 20 years who received health examinations from 2009 to 2012, 3,660,117 received four consecutive annual health examinations. Those with one or more missing data (n = 51,002), AF diagnosis before fourth health examination (n = 17,614), and diagnosed as liver cirrhosis or hepatitis (n = 90,714) were excluded. Finally, 3,500,847 subjects were included in this study (Fig. 1). This study was conducted according to the Declaration of Helsinki and was approved by the Institutional Review Board of Seoul National University Hospital (IRB No. E-2103-139-1206). Also, the informed consent was waived by the Institutional Review Board of Seoul National University Hospital.

### Baseline characteristics and measurements

Body mass index (BMI) was calculated by dividing weight by height squared. Baseline comorbidities including diabetes mellitus, hypertension, dyslipidemia, chronic kidney disease and heart failure were defined by using the ICD-10-CM and prescription codes^9^. Smoking status and alcohol consumption were evaluated by using a standardized self-reporting questionnaire. Smoking status was classified as never smoker, ex-smoker, and current smoker. A heavy drinker was defined as a subject who consumed more than 30 grams of alcohol daily for both men and women.

Newly diagnosed nonvalvular AF was defined as a recording of ICD-10 codes I480-484, and I489 during hospitalization or at least two diagnoses at an outpatient clinic within 1 year. Those with a diagnosis of mitral stenosis (I050, I52, and I059) or having mechanical heart valves (Z952-Z954) were considered to have valvular AF and were excluded. The occurrence of AF was evaluated until December 31, 2018.

### Cumulative GGT score: accumulation of elevated GGT level

There were four GGT data, including baseline and three follow-up tests. Each GGT level was divided by a quartile, and the highest GGT quartile was regarded as point 1. Cumulative GGT score was defined as the number of the highest GGT quartile amongst four examinations (Fig. 1, score 0 to 4). After the last health examination, patients were divided into five groups by a cumulative GGT score from 0 to 4.

### Statistical analysis

Continuous variables were expressed as mean values with their standard deviations, and categorical variables were expressed as numbers and percentages. One-way analysis of variance and the chi-square test were used to evaluate the differences among the groups divided by a cumulative GGT score. The incidence rate of AF was presented per 1000 person-years. Cox proportional hazards regression analysis was performed to estimate adjusted hazard ratios and 95% confidence intervals for AF incidences in the highest 4 cumulative GGT score groups compared to the lowest group. Model 1 was an unadjusted model; data adjustment was performed with two different models. Model 2 was adjusted with age and sex. Model 3 was adjusted with additional covariates, including smoking, alcohol consumption, physical activity, income, hypertension, diabetes mellitus, dyslipidemia, BMI, heart failure and coronary artery disease. A P-value of < 0.05 was considered statistically significant. All statistical analyses were conducted using SAS version 9.3 (SAS Institute Inc., Cary, NC, United States) and R version 3.2.3 (The R Foundation for Statistical Computing, Vienna, Austria). For figures and plots generation, we used Microsoft Excel and PowerPoint version 2306.

## Results

### Baseline characteristics

The baseline characteristics of the study population were summarized in Table 1. The mean age and proportion of male were 42.2 ± 10.9 and 71.9%. The current smokers and heavy drinkers were 33.9% and 7.7%, respectively. Mean AST, ALT and GGT were 25.0 ± 12.0U/L, 26.1 ± 19.3U/L, and 38.7 ± 45.4U/L. The entire population was divided into 5 group according to the cumulative number of the highest GGT: group 0 with 2,135,973 (61.0%), group 1 with 404,570 (11.6%), group 2 with 255,044 (n = 7.3%), group 3 with 246,342 (7.0%), and group 4 with 458,918 (13.1%) (Fig. 1).

**Table 1:** Baseline characteristics of the study population.

	Total	Cumulative GGT score					p-value
		0	1	2	3	4	
	(n=3,500,847)	(n=2,135,973)	(n=404,570)	(n=255,044)	(n=246,342)	(n=458,918)	
Age (years)	42.2 ± 10.9	41.1 ± 11.0	43.0 ± 10.9	43.6 ± 10.6	44.1 ± 10.3	44.9 ± 9.8	<0.0001
Sex (male)	2,518,449 (71.9)	1,571,925 (73.6)	252,629 (62.4)	172,467 (67.6)	176,871 (71.8)	344,557 (75.1)	<0.0001
Current smoker	1,188,261 (33.9)	675,773 (31.6)	124,890 (30.9)	89,920 (35.3)	95,736 (38.9)	201,942 (44.0)	<0.0001
Heavy alcohol consumption	270,257 (7.7)	105,197 (4.9)	31,101 (7.7)	26,150 (10.3)	31,221 (12.7)	76,588 (16.7)	<0.0001
Regular exercise	709,946 (20.3)	434,693 (20.4)	81,513 (20.2)	52,157 (20.5)	51,077 (20.7)	90,506 (19.7)	<0.0001
Income (low 25%)	756,351 (21.6)	422,953 (19.8)	102,780 (25.4)	63,455 (24.9)	59,201 (24.0)	107,962 (23.5)	<0.0001
Diabetes mellitus	215985(6.2)	80991(3.8)	26570(6.6)	21726(8.5)	25674(10.4)	61024(13.3)	<0.0001
Hypertension	657,047 (18.8)	289,355 (13.6)	82,536 (20.4)	62,319 (24.4)	69,343 (28.2)	153,494 (33.5)	<0.0001
Dyslipidemia	509,537 (14.6)	210,546 (9.9)	66,893 (16.5)	51,044 (20.0)	55,946 (22.7)	125,108 (27.3)	<0.0001
Coronary artery disease	76,512 (2.19)	38,098 (1.78)	10,053 (2.48)	7086 (2.78)	7450 (3.02)	13,825 (3.01)	<0.0001
Chronic kidney disease	143,221 (4.1)	87,263 (4.1)	17,221 (4.3)	10,816 (4.2)	10,159 (4.1)	17,762 (3.9)	<0.0001
Heart failure	6991 (0.2)	3288 (0.2)	953 (0.2)	630 (0.3)	728 (0.3)	1392 (0.3)	<0.0001
Body mass index (kg/m^2^)	23.7 ± 3.2	23.1 ± 2.9	23.9 ± 3.2	24.5 ± 3.2	24.9 ± 3.3	25.3 ± 3.3	<0.0001
Systolic BP (mmHg)	121.7 ± 13.6	119.9 ± 13.0	121.8 ± 13.7	123.5 ± 13.8	124.9 ± 13.9	126.8 ± 14.2	<0.0001
Diastolic BP (mmHg)	76.6 ± 9.5	75.4 ± 9.1	76.7 ± 9.5	77.8 ± 9.6	78.8 ± 9.7	80.1 ± 9.9	<0.0001
Fasting glucose (mg/dL)	95.6 ± 20.8	93.0 ± 17.1	95.8 ± 20.7	97.9 ± 23.0	99.8 ± 25.1	103.5 ± 28.6	<0.0001
Total cholesterol (mg/dL)	193.9 ± 34.9	189.1 ± 32.9	196.3 ± 35.1	199.6 ± 35.7	201.9 ± 36.5	206.7 ± 37.8	<0.0001
HDL-cholesterol (mg/dL)	54.6 ± 14.6	54.8 ± 14.3	55.0 ± 15.1	54.3 ± 15.0	53.8 ± 14.8	53.8 ± 14.9	<0.0001
LDL-cholesterol (mg/dL)	112.2 ± 32.1	110.9 ± 30.2	113.7 ± 32.7	114.6 ± 33.8	114.6 ± 34.9	114.5 ± 36.7	<0.0001
eGFR (ml/min/1.73m^2^)	89.3 ± 40.7	89.5 ± 41.7	89.2 ± 39.1	89.1 ± 40.1	89.0 ± 40.0	88.9 ± 38.1	<0.0001
Triglyceride (mg/dL)	137.0 ± 95.0	117.6 ± 75.4	139.5 ± 92.4	155.9 ± 101.8	170.5 ± 110.3	197.0 ± 127.9	<0.0001
AST (U/L)	25.0 ± 12.0	22.5 ± 8.3	25.3 ± 11.6	27.0 ± 12.7	28.8 ± 14.3	33.2 ± 19.0	<0.0001
ALT (U/L)	26.1 ± 19.3	21.3 ± 12.3	27.2 ± 20.7	30.7 ± 22.7	34.0 ± 24.5	40.6 ± 28.3	<0.0001
GGT (U/L)	38.7 ± 45.4	22.1 ± 10.0	35.4 ± 23.3	45.7 ± 28.9	59.5 ± 42.9	103.5 ± 87.1	<0.0001

Data are expressed as the mean±SD, or n (%).

Cumulative GGT score was defined as the number of the highest GGT quartile amongst four examinations.

*BP* blood pressure, *eGFR* estimated glomerular filtration rate, *HDL* high-density lipoprotein, *LDL* low-density lipoprotein, *AST* aspartate aminotransferase, *ALT* alanine aminotransferase, *GGT* γ-Glutamyltransferase.

To evaluate the relationship of accumulation of elevated GGT level and AF incidence, the population was divided into five groups by a cumulative GGT score ranging from 0 to 4. In higher cumulative GGT score groups; age, the proportion of heavy alcohol consumption, prevalence of diabetes, hypertension, dyslipidemia, BMI, blood pressure, level of fasting glucose, total cholesterol, triglyceride, AST, ALT, and GGT were significantly higher than in lower cumulative GGT score groups. The proportion of male and current smoker showed same results except group 0.

### Risk of incident AF according to GGT level

During a median follow up 8.0 years (interquartile range: 6.4–8.4) incident AF was developed in 27,752 (0.8%) patients (incidence rate 1.077 per 1000 person-years). The numbers, crude incidence rates, hazard ratios for risk of incident AF according to the baseline GGT quartile and the cumulative GGT score are described in Tables 2 and 3. The number of incident AF and hazard ratio was higher in the higher quartile group and the higher cumulative GGT score group with or without an adjustment of the covariates.

**Table 2:** Association between baseline serum GGT level and risk of AF.

GGT quartile	AF (n)	IR^†^	HR (95% CI)		
			Model 1	Model 2	Model 3
Q1 (n=866,400)	5117	0.801	1 (Ref.)	1 (Ref.)	1 (Ref.)
Q2 (n=900,631)	6285	0.946	1.182 (1.139, 1.227)	1.148 (1.106, 1.191)	1.079 (1.039, 1.120)
Q3 (n=855,011)	7238	1.147	1.432 (1.382, 1.484)	1.293 (1.248, 1.340)	1.152 (1.110, 1.195)
Q4 (n=878,805)	9112	1.416	1.771 (1.712, 1.833)	1.605 (1.551, 1.661)	1.344 (1.295, 1.396)

Model 1: unadjusted.

Model 2: adjusted for age and sex.

Model 3: adjusted for model 1 + smoking, alcohol consumption, physical activity, income, hypertension, diabetes mellitus, dyslipidemia, body mass index, heart failure and coronary artery disease.

*GGT* γ-Glutamyltransferase, *AF* atrial fibrillation, *IR* incidence rate, *HR* hazard ratio, *CI* confidence interval.

^†^Per 1000 person-years.

**Table 3:** Association between cumulative GGT score and risk of AF.

Cumulative GGT score	AF (n)	IR^†^	HR (95% CI)		
			Model 1	Model 2	Model 3
0 (n=2,135,973)	14,277	0.905	1 (Ref.)	1 (Ref.)	1 (Ref.)
1 (n=404,570)	3271	1.100	1.217 (1.172, 1.264)	1.108 (1.136, 1.226)	1.092 (1.051, 1.135)
2 (n=255,044)	2390	1.276	1.412 (1.352, 1.475)	1.315 (1.260, 1.374)	1.182 (1.131, 1.235)
3 (n=246,342)	2594	1.435	1.588 (1.523, 1.655)	1.425 (1.367, 1.486)	1.248 (1.195, 1.302)
4 (n=458,918)	5220	1.559	1.726 (1.673, 1.782)	1.494 (1.448, 1.543)	1.278 (1.235, 1.322)

Model 1: unadjusted.

Model 2: adjusted for age and sex.

Model 3: adjusted for model 2 + smoking, alcohol consumption, physical activity, income, hypertension, diabetes mellitus, dyslipidemia, body mass index, heart failure and coronary artery disease

*GGT* γ-Glutamyltransferase, *AF* atrial fibrillation, *IR* incidence rate, *HR* hazard ratio, *CI* confidence interval.

^†^Per 1000 person-years.

**Figure 2:**
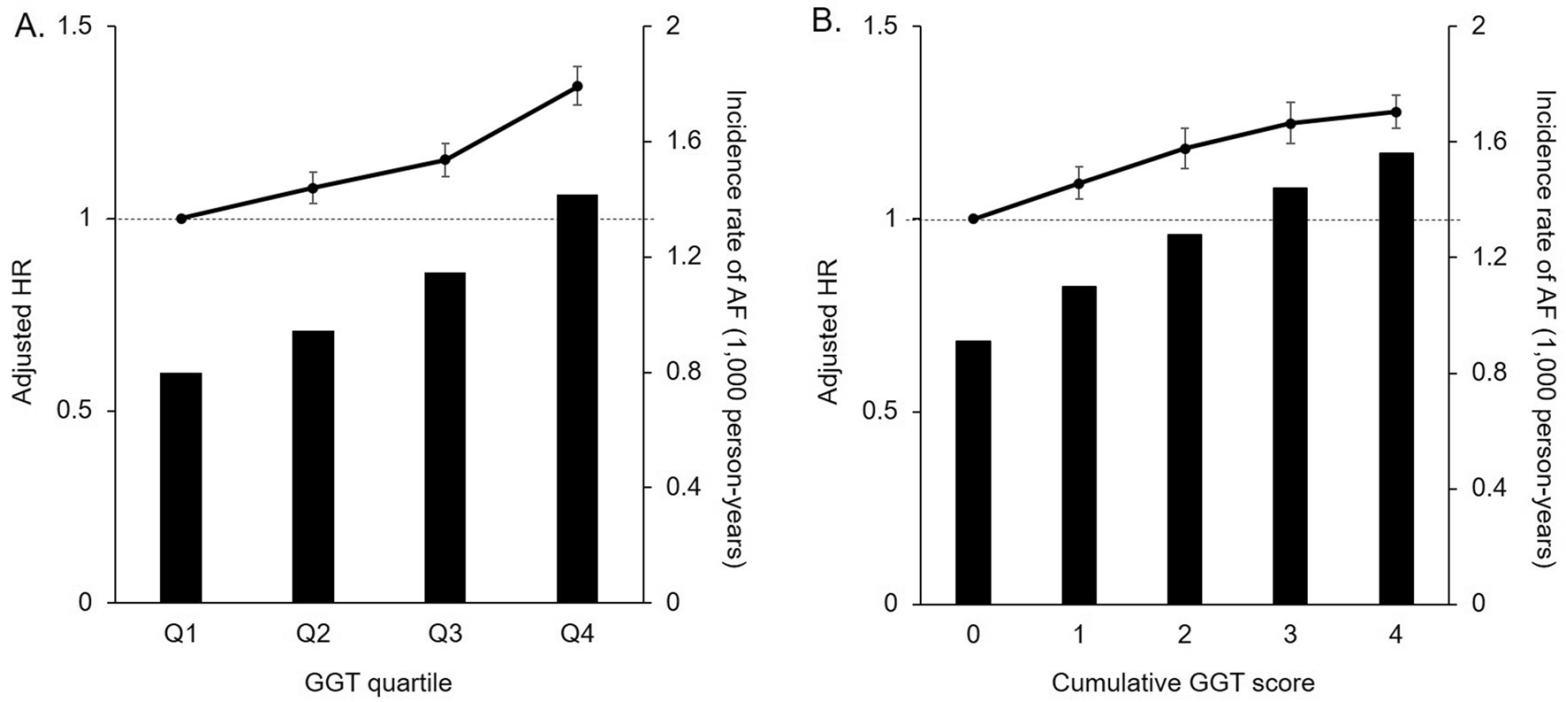
Incident risk of AF based on GGT quartile (**A**) and cumulative GGT score (**B**). After a full adjustment of covariates, the highest quartile showed 33.2% higher risk of AF compared to the lowest quartile (HR 1.332, 95% CI 1.283 to 1.383) and the highest scored group showed 26.5% higher risk of AF than the group that scored 0 (HR 1.265, 95% CI 1.223 to 1.309).

Figure 2 shows the relationship of the risk of incident AF and the level of GGT on the basis of the baseline quartile and cumulative score by adjusted HR. After full adjustment of covariates, the highest quartile showed 34.4% higher risk of AF compared to the lowest quartile (HR 1.344, 95% CI 1.295 to 1.396) and the highest scored group showed 27.8% higher risk of AF than the group that scored 0 (HR 1.278, 95% CI 1.235 to 1.322).

### Subgroup analysis

The subgroup analyses are shown in Table 4. There were no significant interactions among cumulative GGT score, sex, smoking, heavy alcohol consumption, diabetes mellitus, and dyslipidemia on the risk of AF (p for interaction = 0.4669, 0.3003, 0.2033, 0.3101 and 0.5158, respectively).

**Table 4:** Association between cumulative GGT score and risk of AF in subgroups.

Subgroup	Cumulative GGT score	AF (n)	IR^†^	HR (95% CI)		
				Model 1	Model 2	Model 3
Age						
< 65 years	0 (n=2,076,923)	12,027	0.782	1 (Ref.)	1 (Ref.)	1 (Ref.)
	1 (n=392,603)	2775	0.959	1.228 (1.178, 1.280)	1.166 (1.119, 1.216)	1.076 (1.032, 1.122)
	2 (n=247,809)	2063	1.130	1.448 (1.382, 1.517)	1.296 (1.237, 1.358)	1.159 (1.105, 1.215)
	3 (n=239,677)	2294	1.300	1.665 (1.593, 1.741)	1.419 (1.357, 1.484)	1.233 (1.178, 1.291)
	4 (n=447,492)	4645	1.418	1.819 (1.758, 1.882)	1.467 (1.418, 1.518)	1.243 (1.199, 1.290)
≥ 65 years	0 (n=59,050)	2250	5.757	1 (Ref.)	1 (Ref.)	1 (Ref.)
	1 (n=11,967)	496	6.328	1.103 (1.001, 1.215)	1.235 (1.120, 1.363)	1.164 (1.055, 1.286)
	2 (n=7235)	327	6.945	1.211 (1.079, 1.360)	1.395 (1.241, 1.568)	1.291 (1.147, 1.454)
	3 (n=6665)	300	6.917	1.206 (1.069, 1.360)	1.397 (1.237,1.578)	1.272 (1.124, 1.440)
	4 (n=11,426)	575	7.788	1.362 (1.242, 1.492)	1.624 (1.479, 1.784)	1.470 (1.332, 1.621)
*p* for interaction				<0.0001	0.0258	0.0132
Sex						
Male	0 (n=1,571,925)	12,460	1.068	1 (Ref.)	1 (Ref.)	1 (Ref.)
	1 (n=252,629)	2586	1.382	1.295 (1.241, 1.351)	1.202 (1.152, 1.254)	1.104 (1.058, 1.153)
	2 (n=172,467)	1929	1.512	1.417 (1.351, 1.486)	1.340 (1.277, 1.406)	1.199 (1.142, 1.259)
	3 (n=176,871)	2125	1.626	1.523 (1.455, 1.595)	1.439 (1.374, 1.507)	1.258 (1.120, 1.319)
	4 (n=344,557)	4311	1.703	1.599 (1.545, 1.656)	1.505 (1.454, 1.558)	1.289 (1.242, 1.338)
Female	0 (n=564,048)	1817	0.442	1 (Ref.)	1 (Ref.)	1 (Ref.)
	1 (n=151,941)	685	0.622	1.405 (1.287, 1.535)	1.077 (0.986, 1.176)	1.031 (0.944, 1.127)
	2 (n=82,577)	461	0.772	1.747 (1.578, 1.935)	1.191 (1.075, 1.320)	1.101 (0.992, 1.221)
	3 (n=69,471)	469	0.937	2.122 (1.917, 2.348)	1.332 (1.202, 1.476)	1.190 (1.072, 1.321)
	4 (n=114,361)	909	1.111	2.518 (2.325, 2.726)	1.410 (1.300, 1.529)	1.213 (1.113, 1.321)
* p* for interaction				<0.0001	0.1291	0.4669
Current smoker						
No	0 (n=1,460,200)	10,244	0.952	1 (Ref.)	1 (Ref.)	1 (Ref.)
	1 (n=279,680)	2318	1.132	1.190 (1.138, 1.245)	1.094 (1.045, 1.145)	1.093 (1.044, 1.144)
	2 (n=165,124)	1592	1.318	1.386 (1.315, 1.462)	1.163 (1.102, 1.227)	1.167 (1.106, 1.231)
	3 (n=150,606)	1655	1.503	1.581 (1.501, 1.665)	1.224 (1.161, 1.291)	1.228 (1.164, 1.295)
	4 (n=256,976)	3072	1.643	1.730 (1.662, 1.801)	1.234 (1.182, 1.288)	1.249 (1.197, 1.304)
Yes	0 (n=675,773)	4033	0.804	1 (Ref.)	1 (Ref.)	1 (Ref.)
	1 (n=124,890)	953	1.031	1.284 (1.196, 1.378)	1.088 (1.013, 1.169)	1.089 (1.014, 1.170)
	2 (n=89,920)	798	1.200	1.495 (1.386, 1.613)	1.215 (1.124, 1.312)	1.215 (1.125, 1.313)
	3 (n=95,736)	939	1.329	1.655 (1.542, 1.777)	1.287 (1.196, 1.385)	1.287 (1.196, 1.384)
	4 (n=201,942)	2148	1.452	1.813 (1.721, 1.910)	1.316 (1.243, 1.393)	1.326 (1.253, 1.404)
* p* for interaction				0.2400	0.2858	0.3003
Heavy alcohol consumption						
No	0 (n=2,030,776)	13,522	0.902	1 (Ref.)	1 (Ref.)	1 (Ref.)
	1 (n=373,469)	3010	1.098	1.218 (1.171,1.268)	1.182 (1.136, 1.230)	1.100 (1.057, 1.145)
	2 (n=228,894)	2141	1.275	1.416 (1.352,1.482)	1.317 (1.258, 1.378)	1.194 (1.140, 1.251)
	3 (n=215,121)	2259	1.433	1.590 (1.521,1.663)	1.429 (1.366, 1.494)	1.267 (1.211, 1.326)
	4 (n=382,330)	4247	1.524	1.693 (1.636,1.753)	1.465 (1.415, 1.517)	1.276 (1.230, 1.323)
Yes	0 (n=105,197)	755	0.959	1 (Ref.)	1 (Ref.)	1 (Ref.)
	1 (n=31,101)	261	1.130	1.180 (1.025,1.358)	1.057 (0.919, 1.217)	0.994 (0.863, 1.145)
	2 (n=26,150)	249	1.286	1.344 (1.165,1.551)	1.162 (1.006, 1.341)	1.063 (0.920, 1.229)
	3 (n=31,221)	335	1.450	1.515 (1.332,1.723)	1.228 (1.079, 1.396)	1.098 (0.963, 1.252)
	4 (n=76,588)	973	1.731	1.813 (1.649,1.994)	1.415 (1.286, 1.557)	1.247 (1.128, 1.378)
* p* for interaction				0.2848	0.1241	0.2033
Diabetes mellitus						
No	0 (n=2,054,982)	12,897	0.849	1 (Ref.)	1 (Ref.)	1 (Ref.)
	1 (n=378,000)	2787	1.002	1.182 (1.134, 1.231)	1.163 (1.116, 1.211)	1.081 (1.037, 1.127)
	2 (n=233,318)	2016	1.175	1.386 (1.322, 1.452)	1.316 (1.255, 1.379)	1.190 (1.135, 1.249)
	3 (n=220,668)	2079	1.281	1.511 (1.442, 1.582)	1.382 (1.319, 1.448)	1.223 (1.167, 1.283)
	4 (n=397,894)	4125	1.417	1.673 (1.615, 1.732)	1.466 (1.415, 1.518)	1.267 (1.221, 1.316)
Yes	0 (n=80,991)	1380	2.347	1 (Ref.)	1 (Ref.)	1 (Ref.)
	1 (n=26,570)	484	2.522	1.075 (0.969, 1.192)	1.212 (1.093, 1.345)	1.147 (1.033, 1.273)
	2 (n=21,726)	374	2.381	1.015 (0.905, 1.138)	1.206 (1.076, 1.353)	1.134 (1.010, 1.272)
	3 (n=25,674)	515	2.780	1.185 (1.071, 1.312)	1.476 (1.333, 1.635)	1.352 (1.218, 1.501)
	4 (n=61,024)	1095	2.498	1.066 (0.984, 1.154)	1.437 (1.324, 1.558)	1.312 (1.203, 1.431)
* p* for interaction				<0.0001	0.2499	0.3101
Hypertension						
No	0 (n=1,846,618)	9130	0.668	1 (Ref.)	1 (Ref.)	1 (Ref.)
	1 (n=322,034)	1824	0.769	1.152 (1.096, 1.211)	1.160 (1.103, 1.220)	1.108 (1.053, 1.166)
	2 (n=192,725)	1281	0.903	1.353 (1.276, 1.434)	1.304 (1.230, 1.383)	1.219 (1.149, 1.294)
	3 (n=176,999)	1273	0.977	1.463 (1.380, 1.551)	1.352 (1.275, 1.434)	1.251 (1.178, 1.328)
	4 (n=305,424)	2488	1.111	1.666 (1.594, 1.742)	1.463 (1.399, 1.529)	1.332 (1.271, 1.396)
Yes	0 (n=289,355)	5147	2.432	1 (Ref.)	1 (Ref.)	1 (Ref.)
	1 (n=82,536)	1447	2.407	0.991 (0.935, 1.050)	1.089 (1.027, 1.154)	1.055 (0.995, 1.120)
	2 (n=62,319)	1109	2.444	1.006 (0.943, 1.073)	1.158 (1.085, 1.236)	1.115 (1.044, 1.191)
	3 (n=69,343)	1321	2.618	1.078 (1.015, 1.145)	1.277 (1.202, 1.357)	1.212 (1.139, 1.290)
	4 (n=153,494)	2732	2.461	1.014 (0.968, 1.062)	1.256 (1.198, 1.317)	1.182 (1.134, 1.253)
* p* for interaction				<0.0001	0.0017	0.0158
Dyslipidemia						
No	0 (n=1,925,427)	11,885	0.835	1 (Ref.)	1 (Ref.)	1 (Ref.)
	1 (n=337,677)	2476	0.996	1.195 (1.144, 1.248)	1.181 (1.131, 1.234)	1.097 (1.050, 1.146)
	2 (n=204,000)	1767	1.178	1.413 (1.344, 1.485)	1.338 (1.273, 1.407)	1.207 (1.147, 1.269)
	3 (n=190,396)	1851	1.322	1.585 (1.509, 1.665)	1.432 (1.363, 1.504)	1.261 (1.199, 1.326)
	4 (n=333,810)	3581	1.467	1.762 (1.697, 1.829)	1.505 (1.450, 1.562)	1.289 (1.239, 1.342)
Yes	0 (n=210,546)	2392	1.554	1 (Ref.)	1 (Ref.)	1 (Ref.)
	1 (n=66,893)	795	1.630	1.050 (0.969, 1.137)	1.136 (1.048, 1.231)	1.062 (0.979, 1.151)
	2 (n=51,044)	623	1.673	1.077 (0.986, 1.176)	1.202 (1.100, 1.313)	1.100 (1.006, 1.203)
	3 (n=55,946)	743	1.823	1.174 (1.081, 1.275)	1.345 (1.238, 1.461)	1.192 (1.096, 1.297)
	4 (n=125,108)	1639	1.804	1.162 (1.091, 1.238)	1.398 (1.312, 1.490)	1.221 (1.141, 1.306)
* p* for interaction				<0.0001	0.0951	0.5158
Obesity (BMI≥25)						
No	0 (n=1,623,463)	9478,	0.790	1 (Ref.)	1 (Ref.)	1 (Ref.)
	1 (n=264,085)	1911	0.984	1.248 (1.188, 1.311)	1.205 (1.147, 1.266)	1.151 (1.095, 1.209)
	2 (n=148,573)	1297	1.188	1.507 (1.422, 1.597)	1.356 (1.279, 1.437)	1.274 (1.201, 1.351)
	3 (n=148,573)	1296	1.344	1.704 (1.608, 1.806)	1.436 (1.355, 1.522)	1.331 (1.255, 1.413)
	4 (n=219,244)	2401	1.501	1.906 (1.823, 1.994)	1.493 (1.428, 1.562)	1.371 (1.308, 1.437)
Yes	0 (n=512,510)	4799	1.272	1 (Ref.)	1 (Ref.)	1 (Ref.)
	1 (n=140,485)	1360	1.319	1.037 (0.977, 1.102)	1.058 (0.996, 1.124)	1.002 (0.943, 1.065)
	2 (n=106,471)	1093	1.399	1.100 (1.030, 1.175)	1.145 (1.072, 1.223)	1.063 (0.994, 1.136)
	3 (n=114,873)	1298	1.540	1.210 (1.138, 1.287)	1.259 (1.184, 1.339)	1.140 (1.071, 1.214)
	4 (n=239,674)	2819	1.611	1.268 (1.211, 1.329)	1.314 (1.254, 1.377)	1.163 (1.107, 1.223)
* p* for interaction				<0.0001	<0.0001	<0.0001
Any 4 comorbidities^‡^						
No	0 (n=1,324,166)	5613	0.572	1 (Ref.)	1 (Ref.)	1 (Ref.)
	1 (n=191,455)	917	0.650	1.137 (1.061, 1.220)	1.173 (1.094, 1.259)	1.153 (1.075, 1.238)
	2 (n=99,823)	583	0.793	1.387 (1.274, 1.511)	1.340 (1.230, 1.460)	1.305 (1.197, 1.423)
	3 (n=82,425)	537	0.885	1.547 (1.416, 1.691)	1.392 (1.274, 1.521)	1.347 (1.231, 1.473)
	4 (n=123,044)	939	1.041	1.823 (1.701, 1.953)	1.502 (1.401, 1.610)	1.448 (1.348, 1.555)
Yes	0 (n=811,807)	8664	1.451	1 (Ref.)	1 (Ref.)	1 (Ref.)
	1 (n=213,115)	2354	1.507	1.039 (0.993, 1.088)	1.102 (1.053, 1.154)	1.056 (1.009, 1.106)
	2 (n=155,221)	1807	1.589	1.096 (1.042, 1.153)	1.195 (1.135, 1.257)	1.128 (1.071, 1.187)
	3 (n=163,917)	2057	1.713	1.182 (1.126, 1.240)	1.296 (1.235, 1.360)	1.200 (1.142, 1.261)
	4 (n=335,874)	4281	1.750	1.208 (1.165, 1.253)	1.332 (1.283, 1.382)	1.217 (1.170, 1.266)
* p* for interaction				<0.0001	0.0105	0.0003
Metabolic syndrome						
No	0 (n=1,859,457)	10,392	0.756	1 (Ref.)	1 (Ref.)	1 (Ref.)
	1 (n=310,801)	2011	0.879	1.164 (1.110, 1.221)	1.180 (1.125, 1.238)	1.127 (1.074, 1.183)
	2 (n=179,591)	1382	1.046	1.385 (1.309, 1.465)	1.347 (1.274, 1.425)	1.268 (1.198, 1.342)
	3 (n=159,798)	1289	1.096	1.451 (1.369, 1.537)	1.350 (1.274, 1.431)	1.255 (1.183, 1.331)
	4 (n=259,172)	2386	1.256	1.665 (1.593, 1.741)	1.462 (1.399, 1.529)	1.346 (1.285, 1.410)
Yes	0 (n=276,516)	3885	1.916	1 (Ref.)	1 (Ref.)	1 (Ref.)
	1 (n=93,769)	1260	1.841	0.961 (0.902, 1.025)	1.058 (0.993, 1.128)	1.012 (0.949, 1.079)
	2 (n=75,453)	1008	1.830	0.956 (0.892, 1.024)	1.107 (1.032, 1.187)	1.041 (0.970, 1.116)
	3 (n=86,544)	1305	2.067	1.080 (1.014, 1.150)	1.285 (1.206, 1.368)	1.185 (1.111, 1.264)
	4 (n=199,746)	2834	1.955	1.022 (0.974, 1.073)	1.267 (1.206, 1.331)	1.154 (1.095, 1.215)
* p* for interaction				<0.0001	<0.0001	0.0004

Model 1: unadjusted.

Model 2: adjusted for age and sex.

Model 3: adjusted for model 2 + smoking, alcohol consumption, physical activity, income, hypertension, diabetes mellitus, dyslipidemia, body mass index, heart failure and coronary artery disease.

*GGT* γ-Glutamyltransferase, *AF* atrial fibrillation, *IR* incidence rate, *BMI* body mass index.

^†^Per 1000 person-years.

^‡^Hypertension, diabetes mellitus, dyslipidemia, obesity (BMI≥25).

**Figure 3:**
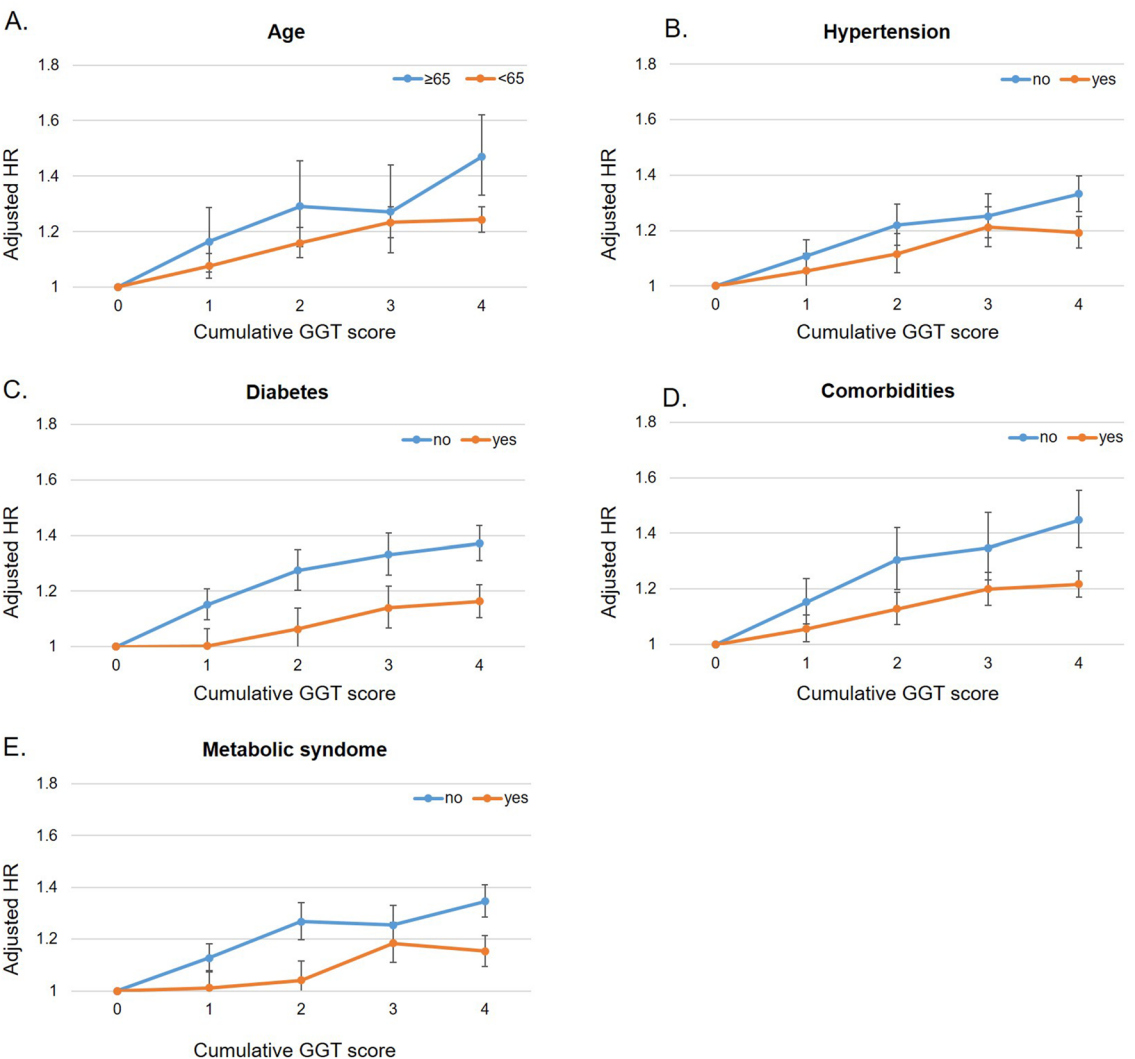
Subgroup analysis for risk of AF according to cumulative GGT score. (**A**) age, (**B**) hypertension, (**C**) diabetes, (**D**) comorbidities (diabetes mellitus, hypertension, dyslipidemia, and obesity), and (**E**) metabolic syndrome. After a full adjustment of covariates, highest scored group showed 46.3% higher risk of AF compared to the group that scored 0 among the elderly people (HR 1.463, 95% CI 1.326 to 1.613), 32.7% higher risk of AF compared to in the group that scored 0 among the people without hypertension (HR 1.327, 95% CI 1.266 to 1.391), 35.8% higher risk of AF compared to the group that scored 0 among the non-obese people (HR 1.358, 95% CI 1.295 to 1.424) and 45.0% higher risk of AF compared to the group that scored 0 among the people without any 4 comorbidities (HR 1.450, 95% CI 1.350 to 1.557).

There were significant interactions among cumulative GGT score, age, hypertension, obesity, any 4 comorbidities (diabetes mellitus, hypertension, dyslipidemia, and obesity) and metabolic syndrome (p for interaction = 0.0132, 0.0158, < 0.0001, 0.0003 and 0.0004, respectively (Table 4 and Fig. 3). After a full adjustment of covariates, the highest scored group showed 47.0% higher risk of AF compared to the group that scored 0 among the elderly people (HR 1.470, 95% CI 1.332 to 1.621), 33.2% higher risk of AF compared to the group that scored 0 among the people without hypertension (HR 1.332, 95% CI 1.271 to 1.396), 37.1% higher risk of AF compared to the group that scored 0 among the non-obese people (HR 1.371, 95% CI 1.308 to 1.437), 44.8% higher risk of AF compared to the group that scored 0 among people without any 4 comorbidities (HR 1.448, 95% CI 1.348 to 1.555) and 34.6% higher risk of AF compared to the group that scored 0 among people without metabolic syndrome (HR 1.346, 95% CI 1.285 to 1.410).

## Discussion

The main findings of our study are as follows: (1) the GGT level and risk of AF have a positive linear correlation; (2) the cumulative GGT score also showed a positive linear correlation with the risk of AF; (3) the positive association was more prominent in the elderly, those without hypertension or metabolic syndrome, non-obese subjects, and the healthy population without diabetes, hypertension, dyslipidemia and obesity.

Recently, the association between the liver enzyme variability and the cardiovascular disease were presented in a large population study^8^. High variability in liver enzymes, including AST, ALT and GGT, was associated with a higher risk of mortality, myocardial infarction, heart failure, and AF. Though the variability of three liver enzymes was associated with AF, only the baseline GGT level was associated with AF. This result was also consistent in a recent meta-analysis of prospective cohort studies regarding liver enzymes and the risk of AF^10^. GGT was significantly associated with the risk of AF, whereas AST and ALT were not.

Also, another study reported that elevated GGT had an independent association with the presence of AF in patients with coronary disease^11^. We have reported a significant association between elevated baseline GGT levels and a risk of AF, especially in a non-obese population^7^.

In a subgroup analysis of our study, the elderly group showed a stronger association of cumulative GGT score and risk of AF than the younger group. This result may come from vulnerability to oxidative stress among the elderly. Ageing is known to have a relationship with decreased antioxidant protection^12–14^. However, with similar accumulation of elevated GGT, the elderly people are at a higher risk of AF than the younger people.

Interestingly, the association between cumulative GGT score and risk of AF was stronger in people without hypertension or obesity. We reported similar results in non-obese subjects^7^. Oxidative stress plays an important role in the pathophysiology of hypertension^15,16^. Obesity itself provokes oxidative stress via increased production of pro-inflammatory adipocytokines^17,18^. Therefore, people with hypertension or obesity might be already exposed to substantial oxidative stress. In these groups, the cumulative GGT score may not fully reflect an additive exposure of oxidative stress. This could weaken the relationship between the cumulative GGT score and the risk of AF. However, those without hypertension and non-obese people showed a stronger association with the cumulative GGT score and risk of AF. In the case of dyslipidemia, a similar trend was observed without statistical significance after the adjustment of covariates.

When subgroup analysis was done with a healthy population without a diagnosis of diabetes mellitus, hypertension, dyslipidemia, and obesity, the risk of AF was most stiffly increased by a higher cumulative GGT score. Subjects without comorbidity may have less exposure to oxidative stress than those with some comorbidities. The result of people without metabolic syndrome was similar in this context. This result suggests that GGT level or cumulative GGT score could be an effective marker for AF risk, especially in healthy populations with few or no comorbidities. As medical science advances, the causes of various diseases are being identified and treatments are also being advanced. In particular, the importance of primary prevention is emerging in modern medicine, and heart diseases are no exception^19^. The relationship between GGT and AF confirmed in this study was particularly important in this context, where the importance of primary prevention is emphasized because healthy subgroup showed more relevance.

Atrial remodeling promotes reentry and ectopic activities, which are fundamental physiological mechanisms of AF^20^. Many experimental and clinical data indicated that oxidative stress is implicated in the pathophysiology of atrial remodeling^21^. Molecular mechanisms, including NOX pathway and mitochondrial dysfunction, lead to electrophysiological and structural change^21–23^. Mechanistic links between oxidative stress and atrial remodeling are complex and still need further evaluation. It is clear that oxidative stress plays a role in the pathophysiology of AF.

There are some limitations to this study. Firstly, this was a retrospective observational study. Therefore, our results cannot confirm the causality between the cumulative GGT score and AF incidence. Secondly, included data was derived from those who got annual health checkups and mainly, those who were employed. This led to a 72% of male proportion, which is far higher than the real proportion of the Korean population. There may be a potential selection bias, which has limitations to represent the entire Korean population. Thirdly, there was no data about medication that could influence liver enzyme levels. Fourthly, the data of inflammatory markers like hsCRP or ESR was not collected. Fifth, in this study, to calculate the GGT score based on the consecutively performed health examination, we include patients who had their index examination between 2009 and 2012 and had 3 more consecutive examinations before that, so we had 4 consecutive examinations. We followed them until December 31, 2018, to ensure we had at least 6 years of follow-up. The limitation in interpreting the results for current medicine may arise from the necessity to rely on historical consecutive examination data to construct a cohort, resulting in a somewhat distant temporal context from the present. Finally, the NHIS database relies on the diagnostic code of AF for the diagnosis of AF. Although there is a possibility of misdiagnosis of AF, the overall incidence of AF in this data (0.8%) was similar to previously reported data^24^.

In conclusion, our study demonstrated the independent association of cumulative GGT score and AF incidence with the dose-response relationship. The cumulative GGT score was more correlated in specific subgroups, such as the elderly, people without hypertension, non-obese people, and especially the healthy population without comorbidities. Our results suggest that multiple measurements of high GGT levels might be a useful marker for risk stratification of AF development, especially in the elderly and healthy population.

## Data Availability

The datasets generated during and/or analysed during the current study are available from the corresponding author on reasonable request.

## Abbreviations

AF: Atrial fibrillation
ALT: Alanine aminotransferase
AST: Aspartate aminotransferase
BMI: Body mass index
GGT: γ-Glutamyltransferase
NHIS: National health insurance service
